# The Effect of Phosphoric Acid on the Flame Retardancy and Interfacial Adhesion of Carbon Fiber with Thermoplastic Resin PA6

**DOI:** 10.3390/polym17030381

**Published:** 2025-01-30

**Authors:** Gyungha Kim, Daeup Kim

**Affiliations:** Carbon & Light Materials Application Group, Korea Institute of Industrial Technology, Jeonju 54853, Republic of Korea; gyunghakim@gmail.com

**Keywords:** carbon fiber, phosphoric acid, interfacial shear strength, functional group, mechanism

## Abstract

This study investigated the effect of phosphoric acid treatment on carbon fibers to enhance their flame retardancy, the impact of carbon fiber surface treatment conditions on the interfacial adhesion between carbon fibers and PA6, and the chemical reaction mechanisms on the carbon fiber surface. Phosphoric acid treatment resulted in a flame-retardant effect, with a limited oxygen index of over 52.8%, and V0 level flame retardancy characteristics in the UL-94 test when the concentration exceeded 0.5 vol.%. When the treatment time was fixed at 30 min, the tensile strength increased by approximately 2%, and the interfacial shear strength (IFSS) increased by approximately 11% at 0.5 vol.% phosphoric acid, accompanied by an increase in hydroxyl (C–O), carbonyl (C=O), phosphate (P–O), and phosphoryl (P=O) groups. However, at concentrations higher than 0.5 vol.%, the tensile strength decreased by approximately 90%, and the IFSS decreased by approximately 12%, compared to the untreated nonwoven fabric. When the treatment time was varied at 0.5 vol.% phosphoric acid, both the tensile strength and IFSS increased continuously up to 10 min, with a 31% increase in tensile strength and a 17% increase in IFSS, along with an increase in O=C–O, P–O, and P=O groups, as well as surface energy. After 10 min, the tensile strength decreased by approximately 20%, while the IFSS and surface energy remained relatively unchanged.

## 1. Introduction

Carbon fibers are lightweight materials with high specific strength, heat resistance, and excellent electrical conductivity and their composites are expected to be applied in various industries, including defense and aerospace [1,2,3,4,5,6,7,8,9]. However, carbon fibers are highly susceptible to oxidation reactions at temperatures above 500 °C, which leads to the deterioration of their thermal and mechanical properties and a reduction in interfacial adhesion. Therefore, carbon fiber composites used in automotive interior components, among other applications, require flame retardancy at the Underwriters Laboratories(UL)-94 V0 level, Necessitating flame-retardant treatments to meet these requirements [10,11]. UL-94 is a standard used to assess the flammability of plastics. The UL-94 flammability ratings include HB, V-2, V-0, and 5V, with flame resistance increasing progressively from left to right.

Generally, methods for the flame-retardant treatment of composites include treating the resin [12,13,14,15,16,17,18,19], composite surface [20,21,22,23,24], and fiber surface [25,26,27,28,29] with flame retardants. Studies on the flame retardancy of carbon fiber composites after treating epoxy resin with phosphorus-based oligomeric flame retardants have shown that the flame retardant contributes to flame suppression and char formation, thereby improving the UL-94 rating of the composites from HB to V0 [14]. Shanjun et al. reported that the flame retardancy of carbon fiber composites was enhanced by treating a polypropylene (PP) resin with ammonium polyphosphate (APP) [30]. The carbon layer formed on the carbon fiber during combustion suppressed the heat transfer to the fiber, resulting in excellent flame retardancy with a V0 rating in the UL-94 test. However, when the resin was treated with flame retardants, the resin in the composite was replaced with a flame retardant, which reduced the cross-linked volume between the fibers and resin. This reduction in volume weakened the interfacial adhesion between the fiber and the resin, and the flame retardant altered the original structure of the resin, reducing the load-bearing components of the material, which in turn decreased the tensile strength [3].

Studies on the treatment of composites with flame retardants have shown that when sisal/PP composites are treated with Mg(OH)_2_ and zinc borate-based flame retardants, the long carbon chains contained in the flame retardant bond to the fibers, reducing the combustion rate and improving the thermal stability of the composite without damaging its tensile and bending properties [19]. Additionally, according to Suppakarn et al., when the surfaces of glass fiber/epoxy composites were treated with expandable and non-expandable char-based flame retardants as well as halogenated flame retardants, the expandable char-based flame retardant provided the greatest improvement in flame retardancy [20]. This was attributed to the expansion of the expandable char under heat, which formed an insulating layer on the exposed surface of the composite, thereby significantly reducing the thermal conductivity compared to the non-expandable char and halogenated flame retardants. In another study, Pomazi et al. reported that when a flame retardant prepared by mixing APP and resorcinol bis-diphenyl phosphate (RDP) was applied to the surface of carbon fiber/epoxy composites under vacuum, the UL-94 rating improved from HB to V0, and the vacuum-treated composites exhibited stronger bonding between the carbon fiber and resin compared to the untreated composites [24]. However, when flame retardants are applied to composites, it is essential that they do not degrade due to external factors such as moisture, tension, or impact. Therefore, in addition to the interfacial adhesion between the fiber and resin, complex treatment processes are required to address issues such as water resistance, weatherability, impact resistance, and lightweight properties.

In contrast, it has been observed that treating kenaf fibers with flame-retardant aluminum trihydroxide (ATH) results in a greater distribution of ATH particles on the fiber surface as the ATH content increases, which roughens the surface and enhances the interfacial adhesion between the kenaf fiber and polylactic acid (PLA) resin. ATH, which absorbs heat and decomposes to release water at 220 °C, plays a cooling role by reducing the polymer surface temperature. As the ATH content increases, the thermal shrinkage and expansion decrease, and the limited oxygen index (LOI) tends to increase [26]. When Muga fibers were treated with the flame retardant APP and evaluated in PLA composites, it was found that the phosphorus present in APP enhanced the thermal stability, resulting in excellent flame retardancy with a V0 rating in the UL-94 test. However, APP was reported to interfere with the interaction between the muga fiber and PLA, causing a reduction in tensile strength by approximately 10% after the flame retardant treatment [27]. After treating the carbon fibers with the flame retardant PU and combining them with zirconium phosphate nanosheets, their flame retardancy was analyzed. The carbon fibers acted as a skeleton, forming a strong and dense char layer. A flame-retardant composite containing approximately 3 wt.% PU-treated zirconium phosphate exhibited a 10% increase in the LOI and a 65% reduction in the peak heat release rate compared to the untreated composite, as reported by Ran et al. [31]. However, flame retardants in the powder form, such as ATH and APP, require high-temperature melting to enhance flame retardancy and interfacial adhesion. When directly applied to fibers, these flame retardants are unevenly distributed, necessitating post-processing at high temperatures, which is a disadvantage.

According to previous studies, although optimal conditions for ensuring the flame retardancy of carbon composites have been suggested when flame retardants are applied to resins and composites, these approaches often lead to increased costs owing to the complex processes [25,26,27,28,29]. Although there are some studies on the direct flame-retardant treatment of carbon fiber surfaces, there is a lack of research on the correlation between flame retardancy and the mechanical properties of carbon composites, and there is insufficient analysis of the underlying causes. This study aimed to improve the flame retardancy of carbon fibers by treating them with phosphoric acid, reviewing their impact on flame retardancy, and analyzing the mechanical and chemical properties of carbon fibers treated with liquid phosphoric acid and thermoplastic resin PA6 to determine the optimal conditions. Additionally, we investigated the effects of phosphoric acid treatment on the oxygen-containing functional groups and the interfacial bonding strength between the carbon fiber and PA6, and elucidated the chemical reaction mechanisms on the surface of the carbon fiber.

## 2. Materials and Experimental Procedure

### 2.1. Materials

T–700 carbon fibers based on PAN, manufactured by Toray, Japan, were used in this study. The mechanical properties are presented as the average values obtained from single-fiber tensile tests in Table 1. Liquid phosphoric acid (85%, Samchun Chemicals, Seoul, Republic of Korea) was used as the flame retardant, and acetone (99.5%, Daejung Chemical & Metals Co., Ltd., Seoul, Republic of Korea) was used for desizing before the flame retardant treatment.

### 2.2. Experimental Methods

The carbon fibers were first desized [32] by immersing them in a beaker containing acetone at 60 °C for 30 min to completely remove the sizing agents on their surface. A round-bottom flask with a reflux condenser was connected, and cold water was circulated to condense and return the acetone vapor generated during heating. After immersion, the fibers were washed three times with distilled water and then dried in an oven at 100 °C for 1 h. The dried carbon fibers were treated with phosphoric acid at concentrations ranging from 0.5 vol.% to 5 vol.% for 30 min at room temperature. Additionally, the fibers were treated with phosphoric acid at a fixed concentration of 0.5 vol.% for a treatment duration of 30 min at room temperature. After immersion, the carbon fibers were washed three times and dried in an oven at 80 °C for 2 h. To prepare the nonwoven carbon fiber fabric for flame-retardancy testing and tensile-strength evaluation, the sizing agents and residual impurities were completely removed from the carbon fibers using the desizing method described above. Then, 2.4 g of the dispersant was dissolved in 500 mL of distilled water using a stirrer at 1000 rpm for 3 h. To the dissolved dispersant solution, 1 g of 12 mm carbon fiber, 1 g of the binder, and 1000 mL of distilled water were sequentially added and stirred for 20 min. The mixture was used to produce a nonwoven fabric using a suction filter, followed by drying at 80 °C for 2 h.

### 2.3. Characteristic Analysis

To analyze the surface morphology and composition of the carbon fibers treated with phosphoric acid, a field emission scanning electron microscope (FE-SEM; JSM-7100F, Jeonju, Republic of Korea) was used, and the analysis was conducted five times for each condition. The flame test was performed for the carbon fibers using an alcohol lamp, with the flame applied to the top of the fibers for 3 min, and each condition was evaluated at least three times. The flame retardancy test of the nonwoven fabric was conducted according to the self-extinguishing combustion test method KS M 458902, and the LOI was analyzed. The nitrogen and oxygen flow rates were adjusted according to the degree of combustion, and the limiting oxygen concentration index was evaluated. Each test condition was measured three times, and the average value was calculated. For thermogravimetric analysis (TGA) of the carbon fibers, a thermogravimetric analyzer was used to analyze the mass changes by heating the sample at a rate of 10 °C/min under nitrogen up to 1000 °C. This was conducted three times for each condition, and the average value was used.

The mechanical properties of the carbon fibers were evaluated using a single-fiber tensile test according to the ASTM D3822 standard with a tensile speed of 5 mm/min. Each condition was tested more than 25 times, and the average value was used. To analyze the interfacial shear strength (IFSS) between the carbon fibers and the PA6 resin, tests were performed according to ASTM C1557 standards, with at least 20 tests for each condition. The carbon fibers were impregnated into the resin at 200 μm and subjected to a pull-out method at a speed of 0.1 mm/min to evaluate the interfacial shear strength. Fourier-transform infrared (FTIR) spectroscopy and X-ray photoelectron spectroscopy (XPS, Thermo Fisher Scientific Inc., Jeonju, Republic of Korea) were used to analyze the changes in the chemical functional groups on the surface of the carbon fibers after phosphoric acid treatment. FTIR was performed by shooting 256 scans at 80 V with KBr pellets, and XPS was conducted using monochromatic Al Kα (1486.6 eV) with high-resolution spectra obtained using a pass energy of 10 eV, step size of 0.05 eV, and 400 μm beam size. To analyze the changes in the surface energy, dynamic contact angle measurements were performed using the Wilhelmy plate method according to ASTM D1331–20. The contact angles were measured using water (hydrophilic) and diiodomethane (99.9%, Sigma-Aldrich Co., LLC, Jeonju, Republic of Korea) (hydrophobic) at an injection rate of 6 mm/min. Each condition was tested at least 20 times, and the average contact angle was used to calculate the surface energy.

## 3. Results and Discussion

### 3.1. The Effect of Phosphoric Acid Treatment on the Flame Retardancy of Carbon Fibers

**Figure 1:**
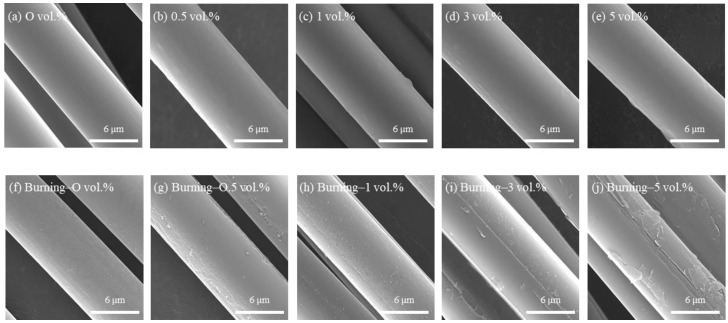
SEM images of carbon fiber treated with various concentrations of phosphoric acid: (**a**–**e**) before flame test and (**f**–**j**) after flame tests.

To evaluate the effect of phosphoric acid treatment on the flame retardancy of carbon fibers, flame tests were conducted on the phosphoric acid-treated carbon fibers and LOI tests were performed on nonwoven fabrics made from carbon fibers. Figure 1 shows a comparison of the surfaces of carbon fibers before and after the flame test, which were treated with phosphoric acid at concentrations ranging from 0.5 to 5 vol.% for 30 min in an atmospheric environment. Before the flame test (Figure 1a–e), the untreated carbon fibers had a smooth surface, whereas after the phosphoric acid treatment, the surface appeared slightly sticky, regardless of the phosphoric acid concentration. After the flame test (Figure 1f–j), the untreated carbon fibers exhibited a smooth surface without any damage, whereas the phosphoric-acid-treated carbon fibers showed an increasing white charred layer on the surface as the concentration of phosphoric acid increased. The surface temperature measured during the flame test was approximately 1200 °C, and the white charred layer is believed to consist of a layer formed by phosphoric acid and oxygen, which are the primary components present in the phosphoric acid treatment.

**Figure 2:**
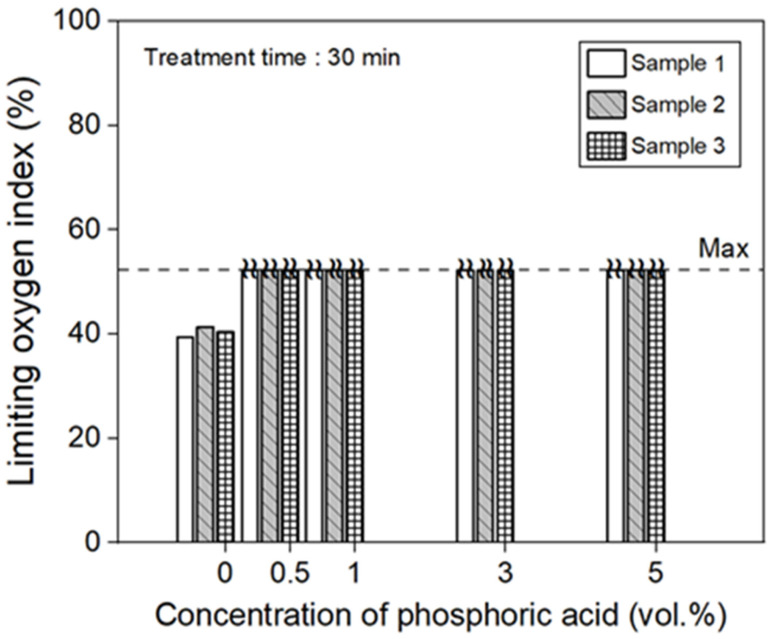
Result of the LOI test of the nonwoven fabric treated with various phosphoric acid concentrations.

LOI tests were conducted to evaluate the flame-retardant properties of the nonwoven fabrics made from phosphoric acid-treated carbon fibers, and the results are shown in Figure 2. The LOI value of the nonwoven fabric without phosphoric acid treatment was between 38 and 40%, whereas those of the phosphoric acid-treated fabric treated for 30 min at concentrations ranging from 0.5 to 5 vol.% (within the scope of this study) reached values above the measurable maximum of 52%. Woo et al. reported that when the LOI values of composite materials treated with 5 vol.%, ATH increased by approximately 6.7% compared to the untreated composites. They explained that when heat is applied to materials containing ATH, a condensation reaction occurs, forming alumina and releasing water, which cools the material and enhances its flame-retardant properties [26]. Shumao et al. reported that a laminated fiber composite treated with 5 wt.% APP showed an approximately 80% increase in the LOI value, reaching 35.6, compared to the untreated composites. This enhancement was attributed to the formation of a char layer on the fiber surface, which was evenly distributed and acted as a thermal barrier. Phosphoric acid, polyphosphoric acid, and non-chlorine gases generated during the high-temperature exposure protected the surface of the fibers and contributed to their flame-retardant properties. This LOI value corresponds to the V0 rating in the UL-94 test [27]. Nannan et al. evaluated the flame retardancy of laminated fibers treated with an amino silane coupling agent and 13.8 wt.% APP. The LOI value increased to 38.9%, reaching a V0 rating in the UL-94 test. This enhancement was attributed to APP acting as an acid source during combustion, generating phosphoric acid, metaphosphoric acid, ammonia, and water. The resulting acids and moisture promoted the formation of char, thereby improving the resistance to combustion [28]. In this study, when the carbon fibers were treated with phosphoric acid at concentrations above 0.5 wt.%, the limited oxygen index (LOI) value exceeded 52%, which, according to previous reports, suggests flame retardancy above the V0 level in the UL-94 test.

**Figure 3:**
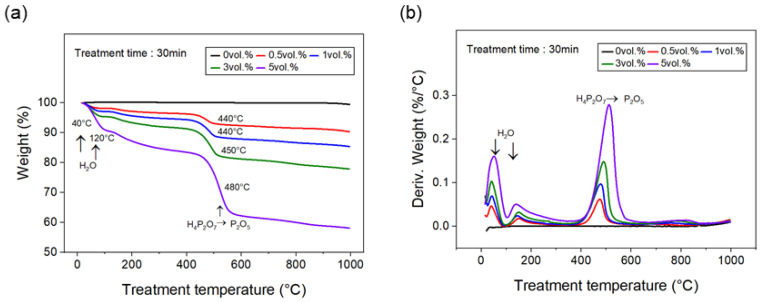
TGA results of carbon fiber treated with various concentrations of phosphoric acid: (**a**) weight loss with increasing treatment temperature, and (**b**) weight loss rate with increasing treatment temperature.

To analyze the white-charred layer remaining on the surface of the carbon fibers after the phosphoric acid treatment, TGA was performed to observe weight changes and phase transitions. TGA graphs of the carbon fibers with varying phosphoric acid concentrations are shown in Figure 3. The weight change and rate of weight change for the carbon fibers showed significant changes at 40 °C and 120 °C, which are believed to result from the removal of residual moisture on the surface of the carbon fibers during phosphoric acid treatment. As the concentration of phosphoric acid increased, the weight change and rate of change at 40 °C and 120 °C also increased. Additionally, at approximately 200 °C, no weight loss was observed; however, phosphoric acid (H_3_PO_4_) was converted to pyrophosphoric acid (H_4_P_2_O_7_), and between 440 °C and 480 °C, pyrophosphoric acid was converted to phosphorus pentoxide (P_2_O_5_), resulting in a weight change. As the concentration of phosphoric acid increased, the weight change, rate of change, and transition temperature increased. At a phosphoric acid concentration of 0.5 vol.%, a weight loss of approximately 4% was observed at 440 °C, while at 5 vol.%, that of approximately 20% occurred at 480 °C. This suggests that the formation of P_2_O) in the 440 °C to 480 °C temperature range reduces heat generation, thus increasing the transition temperature.

Cho et al. evaluated the thermal stability and phase transitions of composite materials with carbon fibers treated with a phosphoric acid solution. They reported that at approximately 200 °C, H_3_PO_4_ was converted into H_4_P_2_O_7_, and at temperatures above 300 °C, it further transformed through (HPO_3_)_n_ to phosphorus. This process formed a protective layer on the fiber surface and improved oxidation resistance. Consequently, the degradation rate of the phosphoric H_2_PO acid-treated composite material at 500 °C was reduced by approximately 79% compared to that of the untreated composite material [11]. Jiang et al. evaluated the flame retardancy of glass fibers treated with the reactive flame retardant 9,10-dihydro-9-oxa-10-phosphaphenanthrene-10-oxide (DOPO) and a silane coupling agent using cone calorimetry. They found that the flame-retardant-treated fibers exhibited a 19.7% reduction in the heat release rate (HRR), a 37.1% reduction in total smoke release (TSR), and a 5.9% decrease in the CO_2_ emission rate compared to untreated fibers. These results confirm that the phosphorus-containing flame retardant generated acid upon heating, and the resulting acid promoted the formation of char, creating a protective barrier on the fiber surface [25]. Shanjun et al. evaluated the thermal stability of carbon composites flame-retarded with 1 vol.% APP and a 0.5 vol.% triazine char-forming agent (TCA) using TGA. They reported that the flame-retarded carbon composite exhibited a 5% increase in the degradation temperature and a 12% greater mass loss than the untreated composites. This was attributed to the consumption of free radicals generated during resin pyrolysis by APP, which interfered with the pyrolysis reaction, whereas APP and TCA combined to form char on the surface of the carbon fiber, thereby enhancing the thermal stability [30]. In the present study, when carbon fibers were flame-retarded with liquid phosphoric acid, it was observed that heating the fibers to temperatures above 200 °C caused a phase transition from H_3_PO_4_ to H_2_PO_4_^−^, and at temperatures between 440 and 480 °C, H_2_PO_4_^−^ transformed into P_2_O_5_. This phase transition is believed to delay the degradation of the carbon fibers, thereby improving their flame retardancy. The results suggest that as the concentration of phosphoric acid increases, the amount of P_2_O_5_ formed at the final stage also increases, leading to a greater delay in degradation. For phosphoric acid concentrations greater than 0.5 vol.%, the composites exhibited an LOI value of 52.8% or higher, and achieved V0-level flame retardancy in the UL-94 test.

### 3.2. The Effect of Phosphoric Acid Treatment on the Interfacial Bonding Strength Between Carbon Fiber and PA6 Resin

**Figure 4:**
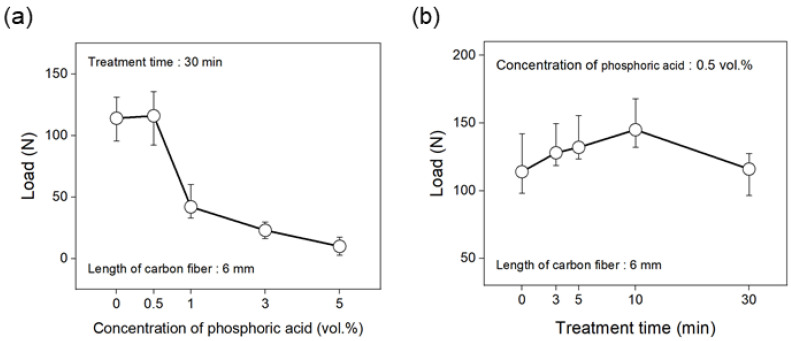
Variation in the tensile strength of the nonwoven fabric according to treatment conditions: (**a**) concentration of phosphoric acid, and (**b**) treatment time.

The tensile strength of the carbon fiber nonwoven fabric as a function of the phosphoric acid concentration and treatment time is shown in Figure 4. To determine the optimal concentration, the treatment time was fixed at 30 min and the tensile strength was evaluated based on the concentration of phosphoric acid. As shown in Figure 4a, at a concentration of 0.5 vol.% phosphoric acid, the tensile strength increased by approximately 2% compared to the untreated nonwoven fabric, but it rapidly decreased at higher concentrations. At 5 vol.%, the tensile strength decreased by approximately 90% (to 10 N) compared to the untreated nonwoven fabric. As shown in Figure 4b, the tensile strength of the nonwoven fabric treated with 0.5 vol.% phosphoric acid continued to increase with treatment time, reaching 145 N at 10 min. However, after 30 min, the tensile strength decreased by approximately 20%, resulting in a tensile strength of 116 N.

Supparkarn et al. reported that adding magnesium hydroxide (Mg(OH)_2_) and zinc borate flame retardants to sisal/PP composites resulted in the flame retardants acting as reinforcing fillers, increasing the tensile strength by approximately 150% compared with that of the untreated composite, to 30 MPa. However, when the Mg(OH)_2_ or zinc borate flame retardants were added individually, the tensile strength of the composite showed little improvement compared to that of the untreated composite because the dispersion of the flame retardants was not uniform [20]. When APP was added to the kenaf/PLA composites, the tensile strength gradually decreased as the APP content increased. For the composites with 50 wt.% APP, the tensile strength was approximately 25% lower compared to the composites without APP. This decrease in tensile strength was attributed to the aggregation of APP, which surrounds the finely chopped kenaf fibers within the PLA matrix, during the compression molding process. This aggregation causes cracks and structural defects, leading to a reduction in tensile strength [26].

**Figure 5:**
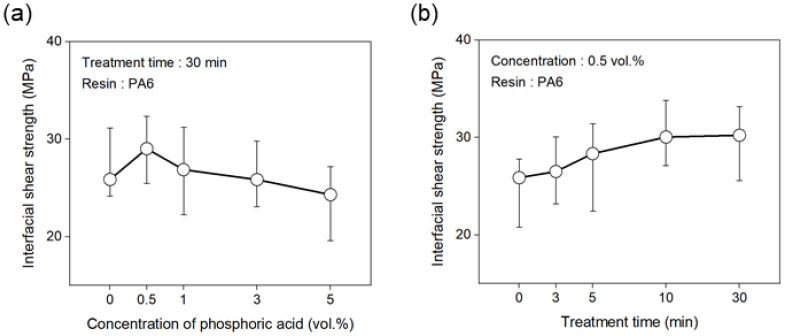
Variation in the IFSS of carbon fibers treated with various conditions: (**a**) concentration of phosphoric acid, and (**b**) treatment time.

Figure 5 shows the interfacial shear strength (IFSS) of the carbon fibers with the PA6 resin under different phosphoric acid treatment conditions to evaluate the interfacial bonding strength. First, the treatment time was fixed at 30 min, and the IFSS was assessed according to the changes in the concentration of phosphoric acid. As shown in Figure 5a, the IFSS of the flame-retarded carbon fibers treated with 0.5 vol.% phosphoric acid increased compared to the untreated carbon fiber, reaching a maximum value of approximately 29 MPa. Above 0.5 vol.%, the IFSS continuously decreased, reaching approximately 24 MPa at 5 vol.%. Furthermore, at an optimal phosphoric acid concentration of 0.5 vol.%, the IFSS increased as the treatment time increased, showing a 17% increase at 10 min compared to the untreated carbon fiber, while there was little change in IFSS at 30 min (Figure 5b). Therefore, the maximum tensile strength and IFSSH were observed under conditions of 0.5 vol. % phosphoric acid for 10 min.

Therefore, in this study, the improvement in the tensile strength of the nonwoven fabric and the interfacial bonding strength between the carbon fiber and PA6 resin during phosphoric acid flame retardant treatment is believed to be due to the formation of cobalt phosphate (Co_3_(PO_4_)_2_) in powder form, generated by the reaction between the carbon in the carbon fiber and the oxygen in the phosphoric acid, as shown in Equation (1) [18]. Cobalt phosphate formed on the surface of the carbon fiber, enhancing the bonding between the carbon fibers in the nonwoven fabric, thereby increasing the tensile strength and IFSS. In contrast, when the phosphoric acid concentration is excessively high, the excessive formation of Co_3_(PO_4_)_2_ on the surface of the carbon fiber is thought to decrease the ability of the nonwoven fabric to withstand load and reduce the interfacial bonding strength between the carbon fibers and the resin.

C + H_3_PO_4_ → C–O/C=O + P_4_ + PO_4_ → Co_3_(PO_4_)_2_ + 3H_2_ (1)

**Figure 6:**
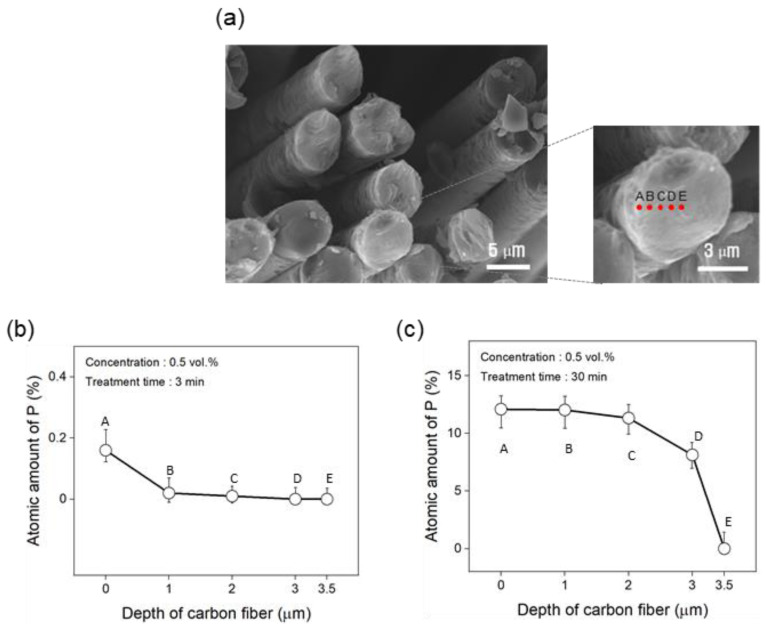
Variation in the atomic amount of phosphorus according to depth (A–E) for the carbon fiber treated with 0.5 vol.% phosphoric acid: (**a**) SEM image, (**b**) 3 min, (**c**) 30 min.

To analyze the cause of the effect of phosphoric acid treatment on the interfacial bonding strength between the carbon fibers and PA6 resin, the amount of phosphorus at different depths of the carbon fibers treated with 0.5 vol.% phosphoric acid for 3 min and 30 min was analyzed (Figure 6). For the carbon fiber flame retardant treated for 3 min with 0.5 vol.% phosphoric acid, phosphorus was only present within 1 μm of the surface of the carbon fiber, and no phosphorus was detected at depths greater than 1 μm (Figure 6a). In contrast, for carbon fibers treated for 30 min, a significant amount of phosphorus was extracted up to a depth of 2 μm, with a slight decrease in phosphorus at a depth of 3 μm, and no phosphorus detected at 3.5 μm (Figure 6b). These results indicate that as the flame-retardant treatment time increased, the amount of phosphorus that penetrated the carbon fiber increased. This suggests that excessive penetration of phosphorus into the carbon fiber may decrease the interfacial bonding strength between the carbon fiber and PA6 resin.

**Figure 7:**
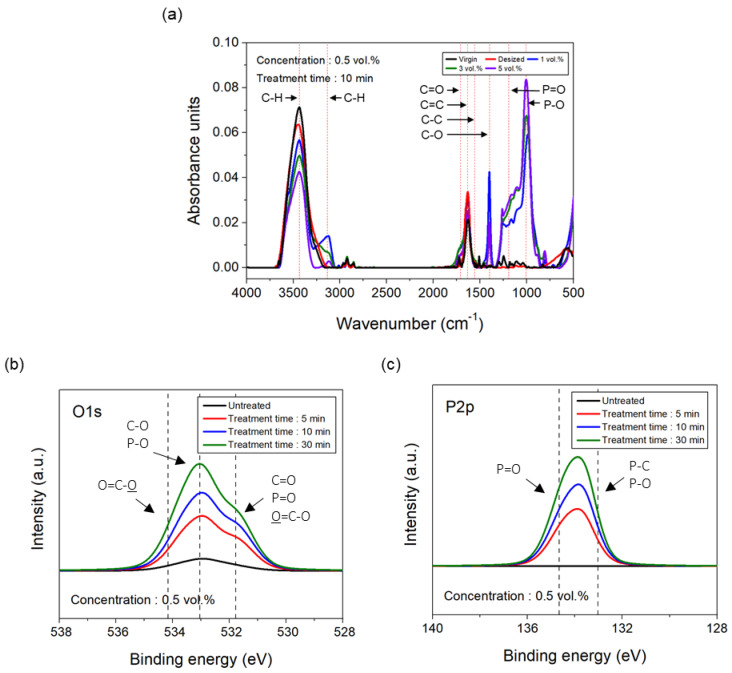
Chemical analysis results of carbon fibers according to the treatment conditions of phosphoric acid: (**a**) FTIR spectra, (**b**) O1s XPS spectra, (**c**) P2p XPS spectra.

Furthermore, to analyze the chemical changes in the interfacial bonding strength caused by the phosphoric acid treatment, the chemical functional groups on the carbon fibers were analyzed using FTIR and XPS, and the results are presented in Figure 7. In the FTIR spectra of the carbon fibers treated with 0.5 vol.% phosphoric acid, as the treatment time increased to 30 min, the number of carbene (C–H, 3480 cm^−1^, 3100 cm^−1^), carbonyl (C=O, 1700 cm^−1^), alkene (C=C, 1600 cm^−1^), alkane (C–C, 1550 cm^−1^), and hydroxyl groups (C–O, 1400 cm^−1^) decreased compared to the untreated carbon fiber, while the number of phosphoryl (P=O, 1230–1250 cm^−1^) and phosphate groups (P–O, 800–1000 cm^−1^) increased (Figure 7a). This suggests that as the phosphoric acid treatment time increased, the number of carbon-carbon and carbon-oxygen bonds within the carbon fiber decreased, whereas the number of oxygen-phosphoric acid bonds increased. Additionally, in the O1s spectra obtained from the XPS analysis (Figure 7b), as the treatment time increased to 30 min, the number of C–O and C=O groups decreased, whereas the number of lactone (O=C–O), P–O, and P=O groups increased compared to the untreated carbon fiber. Furthermore, in the P2p spectra (Figure 7c), the levels of P–O and P=O increased as the treatment time increased to 30 min. This suggests that the C–O and C=O groups on the surface of the carbon fibers combined with the oxygen in phosphoric acid to form O=C–O groups, thus decreasing C–O and C=O while increasing O=C–O. The oxygen and phosphorus present in phosphoric acid likely combine with the oxygen on the carbon fiber surface, resulting in a significant increase in the P–O and P=O bonds. Additionally, to quantitatively assess the changes in the surface composition of the carbon fibers and the increase in the oxygen content, the O/C ratio was calculated, as shown in Table 2. For the carbon fibers treated with 0.5 vol.% phosphoric acid, the amount of carbon and nitrogen decreased with increased treatment time up to 30 min, while the amounts of oxygen and phosphorus significantly increased. Moreover, the O/C ratio for the phosphoric acid-treated carbon fibers continuously increased with treatment time, reaching approximately 16 times higher than that of the untreated carbon fibers after 30 min. This increase, which is much higher than the surface activity threshold of 0.14, indicates that the phosphoric acid treatment increased the amount of oxygen bonded to the surface of the carbon fibers, thereby enhancing the interfacial bonding strength with the PA6 resin [33]. Ran et al. analyzed the changes in the chemical composition of composites by coating carbon fibers with 2.7 wt.% PU and then combining them with the PA46/PPO (polyamide 46/polyphenylene oxide) resin. Using FTIR, Raman, and XPS analyses, they reported that after heat treatment of the PU-coated composite, there was a significant increase in P–O–C bond formation due to the large generation of char compared to the uncoated composite. Additionally, the ID/IG ratio decreased by approximately 40%, and the C–N peak increased from 1.3 to 13.0 [31]. Chen et al. evaluated the FTIR spectra of composites that were flame-retarded by 20% ATH and 20% hexakis-(4-boronic acid-phenoxy)-cyclophosphazene (CP-6B) after heat treatment. They reported that the 20% ATH/20% CP-6B composite released and decomposed more flame-inert gases than the 40% ATH composite, effectively diluting combustible volatile substances and thereby enhancing flame retardancy. Consequently, the absorption peaks increased [19].

To examine the change in the surface free energy with respect to the phosphoric acid treatment time, dynamic contact angles were measured at a phosphoric acid concentration of 0.5 vol.%. The polar and nonpolar surface free energies were then calculated by substituting the measurements into Equation (2) [28]:

(2) $$\frac{\gamma_{L}(1+\cos\theta )}{2{\left(\gamma_{L}^{D}\right)}^{\frac{1}{2}}}\;=\;{\left(\gamma_{S}^{P}\right)}^{\frac{1}{2}}\;\times \;\;{\left(\frac{\gamma_{L}^{P}}{\gamma_{L}^{D}}\right)}^{\frac{1}{2}}+{\left(\gamma_{S}^{D}\right)}^{\frac{1}{2}}$$

where $\gamma_{L}$ is the total surface energy of the wetting liquid, $\gamma_{L}^{D}$ is the nonpolar surface energy of the wetting liquid, $\gamma_{L}^{P}$ is the polar surface energy of the wetting liquid, $\gamma_{S}$ is the total surface energy of the specimen, $\gamma_{S}^{D}$ is the nonpolar surface energy of the specimen, $\gamma_{S}^{P}$ is the polar surface energy of the specimen, and θ is the contact angle. The advancing angles of the specimen in contact with the hydrophilic and hydrophobic wetting liquids were substituted into Equation (2), and the polar surface energy of the specimen (slope) and non-polar surface energy (Y-intercept) were obtained from the two coordinates.

**Figure 8:**
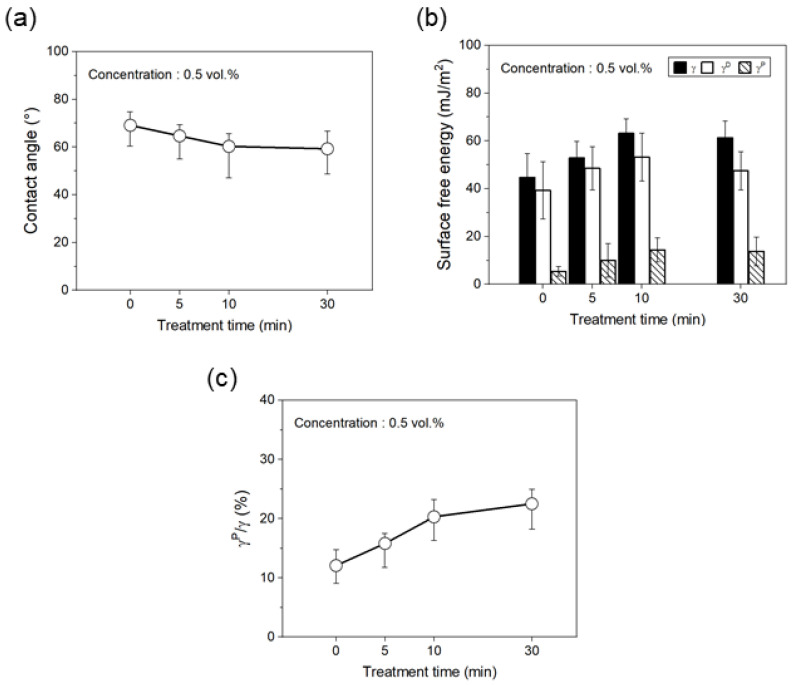
Surface energy of the carbon fiber according to the treatment time of phosphoric acid: (**a**) contact angle, (**b**) surface free energy, and (**c**) ratio of polar surface energy and total surface free energy.

Figure 8 shows the changes in the dynamic contact angle, total surface energy (γ), polar surface energy ($\gamma^{P}$), non-polar surface energy ($\gamma^{D}$), and the ratio of polar surface energy to total surface energy ($\gamma^{P}/\gamma$) of carbon fibers treated with phosphoric acid for different times. The contact angles of carbon fibers treated with 0.5 vol.% phosphoric acid continuously decreased until 10 min, showing a reduction of approximately 13% compared to untreated carbon fiber; after 10 min, the decrease was minimal, within the error range. Additionally, γ and $\gamma^{P}/\gamma$ values increased until 10 min, with the γ value increasing by about 41% and the $\gamma^{P}/\gamma$ value increasing by about 68% compared to untreated carbon fiber. After 10 min, the γ value and $\gamma^{P}/\gamma$ value showed similar values to those of the 10-min treated carbon fiber, within the error range. From these results, it can be concluded that when carbon fiber is treated with phosphoric acid, the oxygen functional groups in the phosphoric acid are significantly introduced to the carbon fiber up to 10 min, leading to a decrease in the contact angle and an increase in γ and $\gamma^{P}/\gamma$ values, which enhances the interfacial adhesion. After 10 min, the amount of oxygen functional groups introduced from phosphoric acid becomes minimal, and the changes in contact angle, γ, $\gamma^{P}/\gamma$, and interfacial adhesion are similar to those of the 10-min-treated carbon fiber, within the error range. Therefore, in this study, when carbon fiber was treated with liquid phosphoric acid, the carbon and oxygen present on the carbon fiber surface reacted with the phosphorus and oxygen in the phosphoric acid. As the treatment time increased, the extent of reaction gradually increased, leading to a continuous decrease in C–O and C=O on the carbon fiber surface and a sustained increase in O=C–O, P–O, and P=O. This increase in the number of oxygen functional groups is thought to enhance the surface energy of the carbon fiber and improve the interfacial adhesion with the PA6 resin.

### 3.3. Chemical Mechanism of Phosphoric Acid Treatment for Enhancing the Interfacial Adhesion of Carbon Fiber

**Figure 9:**
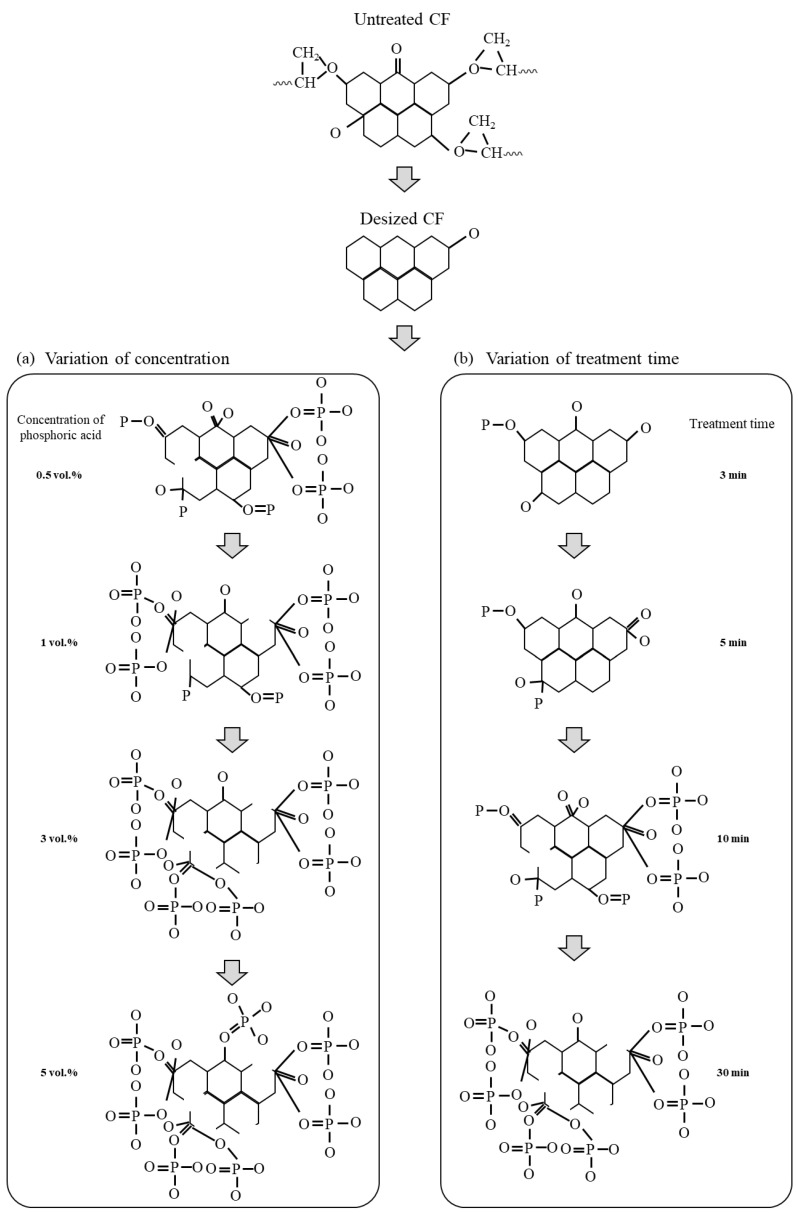
Schematic of the chemical reactions of carbon fiber under different treatment conditions: (**a**) concentration of phosphoric acid and (**b**) treatment time.

Based on the mechanical and chemical properties under different phosphoric acid treatment conditions, the chemical structure and oxygen functional group mechanisms on the carbon fiber surface, as influenced by the phosphoric acid concentration and treatment time, are schematized and presented in Figure 9. First, to examine the effects of phosphoric acid on the carbon fiber surface, the epoxy-based sizing agent coated on the carbon fiber surface was removed by a desizing treatment using acetone. During the desizing process, the oxygen atoms of the hydroxyl group (C–O) and carbonyl group (C=O) present on the carbon fiber surface are bonded with the oxygen atoms in acetone, as shown in Equation (3), resulting in the removal of C–O and C=O as O_2_ and CO_2_.

C–O/C=O + O → C + O_2_↑ + CO_2_↑ (3)

After desizing, the treatment time was initially fixed at 30 min, and the effect of varying the phosphoric acid concentration was evaluated. The results showed that carbon fibers treated with 0.5 vol.% phosphoric acid, there was an increase in hydroxyl (C–O), carbonyl (C=O), phosphate (P–O), and phosphoryl (P=O) groups compared to the desized carbon fibers. However, at the concentration of 1 vol.%, C–O and C=O bonded with phosphoric acid, leading to a decrease in these groups, while the amounts of O=C–O, P–O, P=O, and Co_3_(PO_4_)_2_ increased. At a concentration of 3 vol.%, phosphoric acid continued to bond with the carbon fiber surface, further decreasing C–O and C=O and increasing O=C–O, P–O, P=O, and Co_3_(PO_4_)_2_. At a concentration of 5 vol.%, C–O and C=O slightly decreased, and P–O, P=O, and Co_3_(PO_4_)_2_ showed a minimal increase. These findings suggest that up to a concentration of 0.5 vol.%, the oxygen and phosphorus in phosphoric acid strongly bond with the carbon in the carbon fiber, increasing the interfacial adhesion between the carbon fiber and PA6 resin. However, at concentrations greater than 1 vol.%, excessive phosphorus from the phosphoric acid disrupts the bond between the carbon and oxygen in the carbon fiber, leading to excessive formation of Co_3_(PO_4_)_2_ on the surface, which gradually decreases the interfacial adhesion.

However, when the phosphoric acid concentration was fixed at 0.5 vol.% and the treatment time was varied, the carbon fiber treated for 3 min showed an increase in C–O, C=O, P–O, and P=O bonds, due to the combination of carbon and oxygen from phosphoric acid within the carbon fiber. The carbon fiber treated for 5 min exhibited continuous strong bonding of phosphoric acid to the C–O and C=O bonds on its surface, resulting in a decrease in the C–O and C=O bonds and an increase in the O=C–O, P–O, and P=O bonds. The carbon fibers treated for 10 min showed continued strong bonding of phosphoric acid to the surface, leading to a further reduction in the C–O and C=O bonds and a significant increase in the O=C–O, P–O, and P=O bonds. Additionally, the phosphorus from the phosphoric acid combined with the C–O and C=O bonds of the carbon fiber to form Co_3_(PO_4_)_2_. Furthermore, the carbon fiber treated for 30 min showed excessive formation of Co_3_(PO_4_)_2_ on the surface due to the continued bonding of excess phosphorus from phosphoric acid with carbon and oxygen in the carbon fiber. From these results, it can be inferred that the carbon fibers treated with 0.5 vol.% phosphoric acid for 10 min exhibited a significant increase in oxygen-containing functional groups on the surface, leading to the highest interfacial bonding strength between the carbon fiber and PA6 resin. After 10 min, the oxygen-containing functional groups began to convert into Co_3_(PO_4_)_2_, causing a decrease in the interfacial bonding strength.

## 4. Conclusions

This study aimed to enhance the flame retardancy of carbon fibers by treating them with phosphoric acid and to investigate the effects of phosphoric acid treatment on flame retardancy, interfacial bonding strength between carbon fibers and PA6, and chemical reaction mechanisms on the carbon fiber surface. When the phosphoric acid concentration exceeded 0.5 vol.%, the carbon fibers exhibited flame retardancy of UL-94 V0 or higher. This was attributed to the phase transition from H_3_PO_4_ to H_2_PO_4_^−^ upon heating the carbon fiber surface above 200 °C, and the subsequent formation of P_2_O_5_ from H_2_PO_4_^−^ at 440–480 °C, which ultimately enhanced the degradation delay effect.

1. When the treatment time of phosphoric acid was fixed at 30 min and the effect of phosphoric acid concentration was examined, the results revealed that, compared to the untreated nonwoven fabric, at 0.5 vol.%, the tensile strength increased by approximately 2%, and the interfacial shear strength (IFSS) increased by about 11%. Additionally, the concentrations of hydroxyl group (C–O), carbonyl group (C=O), phosphate group (P–O), and phosphoryl group (P=O) showed an increase. However, at higher concentrations, these values decreased significantly, with the tensile strength at 5 vol.% showing a reduction of approximately 90%, and the IFSS decreasing by about 12%. Moreover, the levels of C–O and C=O decreased substantially, while the concentrations of O=C–O, P–O, P=O, and Co_3_(PO_4_)_2_ increased markedly.
2. When the concentration of phosphoric acid was fixed at 0.5 vol.% and the effect of treatment time was investigated, the results showed that, compared to the untreated nonwoven fabric, the tensile strength increased by approximately 31% and the IFSS increased by approximately 17% for up to 10 min of treatment. The concentrations of O=C–O, P–O, and P=O also increased. However, at 30 min, the tensile strength decreased by approximately 20% compared to the maximum tensile strength observed, and the IFSS and surface energy values remained similar within the experimental error.

## Figures and Tables

**Table 1:** Mechanical properties of carbon fiber.

Mechanical Properties	Carbon Fiber (CF)
Tensile strength (GPa)	4.49
Modulus (GPa)	261
Elongation (%)	2.62
Density (g/cm^3^)	1.80

**Table 2:** Surface elemental composition of carbon fibers according to the treatment time of phosphoric acid.

Treatment Time	Elemental Composition (at.%)				O/C
	C1s	O1s	N1s	P2p	
O	83.83	12.46	3.51	–	0.15
5 min	30.94	52.28	3.40	13.38	1.68
10 min	28.09	53.79	3.31	14.81	1.91
30 min	22.98	58.52	2.33	16.17	2.54

## Data Availability

Data are contained within the article.
